# Supplementary material for: Fast Synthesis of Au Nanoparticles on Metal–Phenolic Network for Sweat SERS Analysis
Source: Nanomaterials (Basel). 2022 Aug 28;12(17):2977. doi: 10.3390/nano12172977 (PMC9458096; doi:10.3390/nano12172977)
Supplement: Supplementary file 1 [file nanomaterials-12-02977-s001.zip › nanomaterials-1835650-supplementary.pdf]

Supplementary Materials

Fast Synthesis of Au Nanoparticles on Metal–Phenolic Network for Sweat SERS Analysis

Xiaoying Zhang [1], Xin Wang [2], Mengling Ning [3], Peng Wang [2], Wen Wang [2], Xiaozhou Zhang [4], Zhiming Liu [3,*], Yanjiao Zhang [4,*] and Shaoxin Li [5,*]

1 Department of Physical Education, Guangdong Medical University, Dongguan 523808, China
2 School of Medical Technology, Guangdong Medical University, Dongguan 523808, China
3 Guangzhou Key Laboratory of Spectral Analysis and Functional Probes, College of Biophotonics, South China Normal University, Guangzhou 510631, China
4 School of Basic Medicine, Guangdong Medical University, Dongguan 523808, China
5 School of Biomedical Engineering, Guangdong Medical University, Dongguan 523808, China
* Correspondence: liuzm021@126.com (Z.L.); yjzhang@gdmu.edu.cn (Y.Z.); lishaox@163.com (S.L.)

Citation: Zhang, X.; Wang, X.; Ning, M.; Wang, P.; Wang, W.; Zhang, X.; Liu, Z.; Zhang, Y.; Li, S. Fast Synthesis of Au Nanoparticles on Metal–Phenolic Network for Sweat SERS Analysis. *Nanomaterials* **2022**, *12*, 2977. https://doi.org/10.3390/nano12172977

Academic Editors: Maurizio Muniz-Miranda and Onofrio M. Maragò

Received: 11 July 2022
Accepted: 26 August 2022
Published: 28 August 2022

Publisher's Note: MDPI stays neutral with regard to jurisdictional claims in published maps and institutional affiliations.

Copyright: © 2022 by the authors. Submitted for possible open access publication under the terms and conditions of the Creative Commons Attribution (CC BY) license (https://creativecommons.org/licenses/by/4.0/).

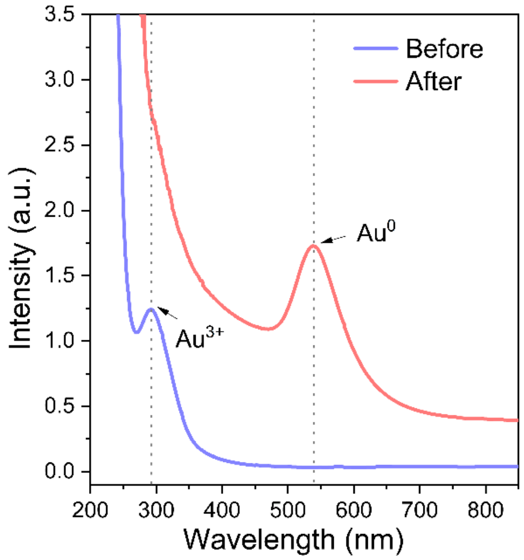

Figure S1. UV-VIS spectra of Au-MPN before and after preparation.

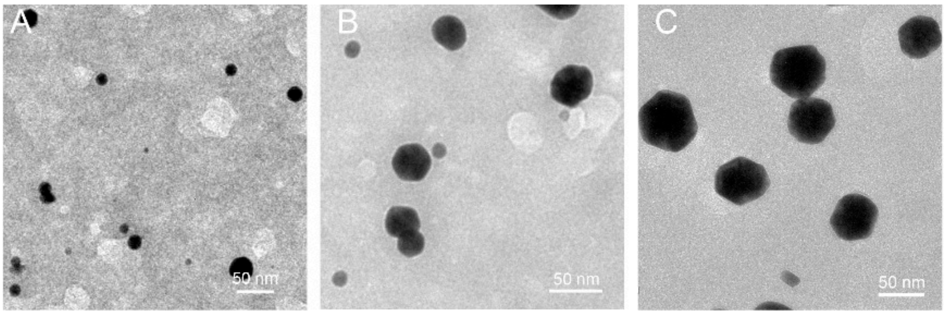

Figure S2. TEM images of (A) Au-MPN-1, (B) Au-MPN-2 and (C) Au-MPN-4, respectively.

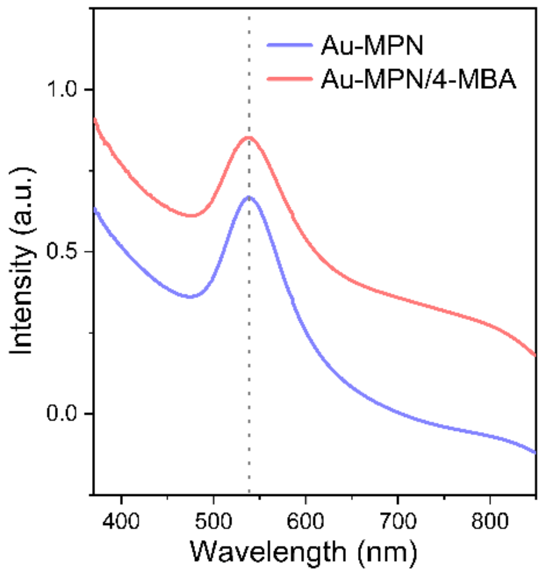

Figure S3. UV-VIS spectra of Au-MPN before and after 4-MBA grafting.

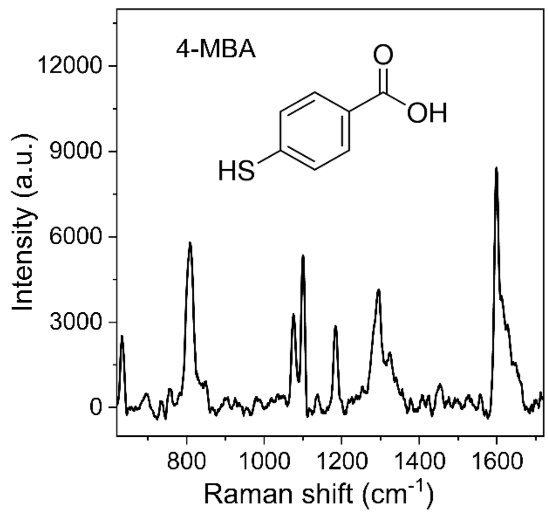

Figure S4. Normal Raman spectrum of 4-MBA (100 mM).
